# MVDA: a multi-view genomic data integration methodology

**DOI:** 10.1186/s12859-015-0680-3

**Published:** 2015-08-19

**Authors:** Angela Serra, Michele Fratello, Vittorio Fortino, Giancarlo Raiconi, Roberto Tagliaferri, Dario Greco

**Affiliations:** 1 NeuRoNe Lab, Department of Computer Science, University of Salerno, Fisciano, Italy; 2 Department of Medical, Surgical, Neurological, Metabolic and Ageing Sciences, Second University of Napoli, Napoli, Italy; 3 Unit of Systems Toxicology and Nanosafety Research Centre, Finnish Institute of Occupational Health, FIOH, Helsinki, Finland

**Keywords:** Clustering, Multi-view, Subclasses

## Abstract

**Background:**

Multiple high-throughput molecular profiling by omics technologies can be collected for the same individuals. Combining these data, rather than exploiting them separately, can significantly increase the power of clinically relevant patients subclassifications.

**Results:**

We propose a multi-view approach in which the information from different data layers (views) is integrated at the levels of the results of each single view clustering iterations. It works by factorizing the membership matrices in a late integration manner. We evaluated the effectiveness and the performance of our method on six multi-view cancer datasets. In all the cases, we found patient sub-classes with statistical significance, identifying novel sub-groups previously not emphasized in literature. Our method performed better as compared to other multi-view clustering algorithms and, unlike other existing methods, it is able to quantify the contribution of single views on the final results.

**Conclusion:**

Our observations suggest that integration of prior information with genomic features in the subtyping analysis is an effective strategy in identifying disease subgroups. The methodology is implemented in R and the source code is available online at http://neuronelab.unisa.it/a-multi-view-genomic-data-integration-methodology/.

**Electronic supplementary material:**

The online version of this article (doi:10.1186/s12859-015-0680-3) contains supplementary material, which is available to authorized users.

## Background

Stratifying patients into distinct subgroups can lead to more accurate diagnostic and treatment strategies. Current methods for patient stratification are usually based on gene expression data and apply cluster algorithms to identify groups of patients having similar expression profiles [1–3]. For example, multivariate gene expression signatures have been shown to discriminate between disease subtypes, such as recurrent and non-recurrent cancer types or tumour progression stages [4]. In addition to gene expression data other omics data types, such as miRNA (microRNA) expression, methylation or copy number alterations, can be used to improve the model accuracy for patient stratification. For example, somatic copy number alterations provide good biomarkers for cancer subtype classification [5]. Data integration approaches to efficiently identify subtypes among existing samples has recently gained attention. The main idea is to identify groups of samples that share relevant molecular characteristics. Strategies of data integration of multiple omics data types poses several computational challenges, as they deal with data having generally a small number of samples and different pre-processing strategies for each data source. Moreover, they have to cope with redundant data as well as the retrieval of the most relevant information contained in the different data sources.

Methods for clustering multiple data layers can be grouped into three main categories, namely early, intermediate, and late integration. Early integration methods directly combine all features into a single dataset [6–8]; intermediate integration methods build joint representations of data given the views [9]; late integration methods preprocess separately each individual view, subsequently combining the results [10, 11]. Late integration methods are often preferred when combining continuous and discrete data together, such as CNV and mRNA. Omics data are highly dimensional data and subject to non-Gaussian noise. Therefore, integrating them with an early or intermediate integration techniques may lead to highly noisy patterns unless appropriate regularization techniques are used which, however, lead to a very complex multi-view learning process.

A number of data integration approaches for patients subgroups discovery were recently proposed, based on supervised classification, unsupervised clustering or biclustering. These methodologies are called multi-view learning [12]. Examples of supervised approaches are [13, 14]. Multi-view biclustering has been used in a cocaine user subtyping [15]. Finally multi-view clustering methodologies have been intensively used also if in few cases on omics data. Multi-view clustering applied to biological data includes iCluster [16] and SNF [9]. iCluster uses a joint latent-variable model to identify the grouping structure in multi omics data. On the other hand, SNF uses a network-based approach to combine different omics data (e.g., mRNA expression, DNA methylation and microRNA expression data) to identify relevant patient subtypes. However, the contribution of the individual data sources to the classification output is not quantified in any of these multi-view clustering methods.

**Fig. 1:**
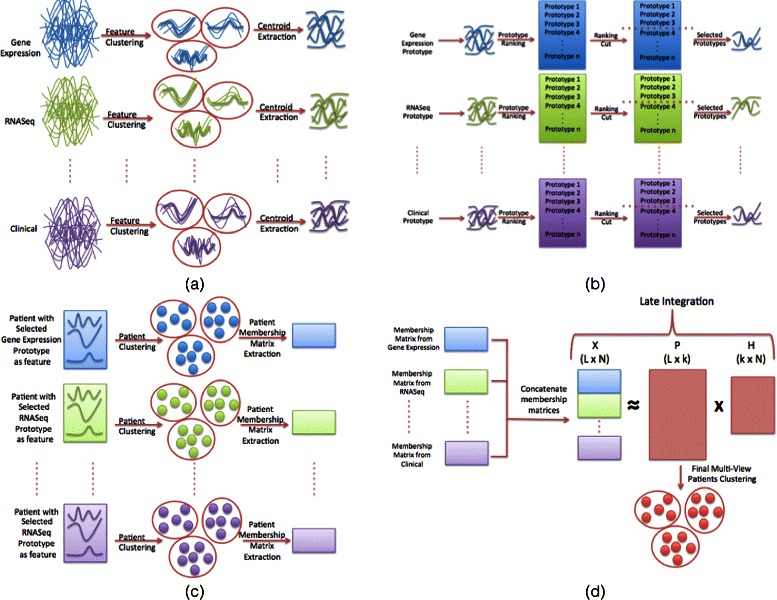
The proposed approach: The computational approach is composed of four steps. First, the data is pre-processed. In each view feature with low variance are filtered out. Furthermore, the features are clustered in order to reduce the input dimension. From each cluster prototype are extracted. These prototypes are the only features used in following steps (**a**). Second, the prototypes are ranked by the patient class separability and the most significant ones are selected (**b**). Third, the patients are clustered and the membership matrices are obtained (**c**). Fourth, a late integration approach is utilized to integrate clustering results (**d**).

In this study, we propose a new computational framework for multi-view clustering that aims to combine dimensional reduction, variable selection, clustering (for each available data type) and data integration methods to find patient subtypes, as described in Fig. 1.

First, the cluster-based correlation analysis is used to reduce the number of features for each data type (genes, miRNAs, protein, etc.). Second, a ranked-based method is employed to select the features based on their ability to separate patient subtypes. Third, clustering is used to identify patient subtypes independently from each reduced dataset. Fourth, integrative clustering methods are exploited to find more robust patient subtypes and assess the contributions of different data types used for the identification of all the patient subtypes. Detailed information on each step can be found in Additional files 1 and 2. We tested our method on large genomic data sets including different omics data types, such as the Cancer Genome Atlas (TCGA) data sets (http://cancergenome.nih.gov/). Our comparison experiments suggest that our method outperforms other existing integration methods, such as Tw-Kmeans [7] and SNF [9].

## Results and Discussion

We developed a novel methodology for cluster analysis of multiple genomic data types.

We compared it with recently developed methods: the integrative clustering algorithm, namely SNF [9]. and the Tw-Kmeans [7], an early integration multi-view clustering model. Using TCGA datasets from 4 different tumor types (Table 1), we evaluated the cluster impurity error, the Normalized Mutual Information [17] and the cluster stability of all the considered algorithms.

**Table 1:** Datasets: Description of the datasets used in this study

Dataset	Response	N(0)	N(1)	N(2)	N(3)	Gene	RNASeq	microRNA	miRNASeq	Protein	Copy	Clinical
						expression		expression		expression	number	data
Breast Cancer from The Cancer genome Atlas, N = 151												
TCGA.BRC	Pam50 (Her2,Basal,LumA,LumB)	24	13	55	59		x		x			
Breast Cancer from The Gene Expression Omnibus, N = 201												
OXF.BRC.1	Pam50 (Her2,Basal,LumA,LumB)	26	6	117	52	x		x				
OXF.BRC.2	Clinical (Level1, Level2, Level3, Level4)	73	54	42	32	x		x				
Prostate Cancer from Memorial Sloan-Kettering Cancer Center, N = 88												
MSKCC.PRCA	Tumor stages T1 vs. T2, T3, T4	53	35			x		x			x	x
Ovarian Cancer from The Cancer Genome Atlas, N = 398												
TCGA.OVG	Tumor stage I,II, Tumor stage III, Tumor stage IV	33	315	50		x		x		x		
Glioblastoma Multiforme from The Cancer genome Atlas, N = 167												
from TCGA.GBM	(Classical, Mesechymal, Neural, Proneural)	37	54	24	52	x		x				

“N” is the number of subjects for each dataset. Ni is the number of samples in the i-th class. An x denotes if that view (column) is available for a specific dataset (row)

The evaluation metrics computed for each dataset are summarized in Table 3. Our unsupervised method shows a mean error of 27,47 %, normalized mutual information (NMI) of 28 % and stability of 85 %. Moreover, the error can significantly decrease when using prior information. Indeed, our method with prior information reduces the error to 6,30 %. The other methods used in the comparison study show a higher mean error from the lowest 30,83 % of SNF to the highest 30,93 % of Kmeans. They also show a lower NMI (the maximum value reached is 26 % of Ward’s method) and variable stability from the lowest 51 % of the Kmeans to the highest 96 % of the partitioning around medoids (pamk).

**Fig. 2:**
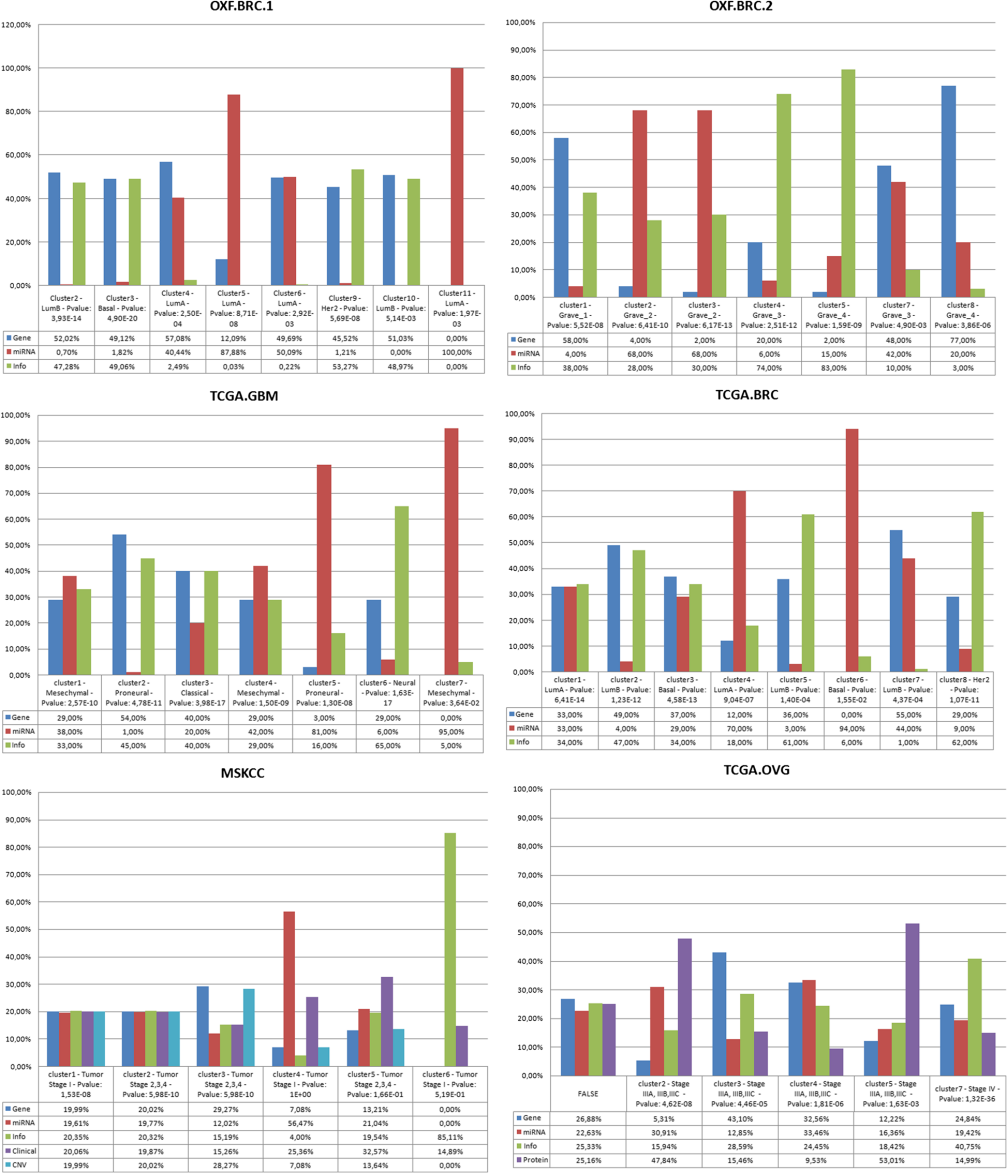
Multi-View Clusters Statistics: For each cluster class label, the p-value and the view contribution are reported. For all the six datasets, the results showed that the matrix factorization method gives lower classification error and better accuracy than the approach with general linear integration

The class label and the p-value for each cluster obtained after the integrative step is reported in Fig. 2, where the label indicates the subclass to which patients in the cluster belong, while the p-value measures the statistical significance of a cluster. In the case of the dataset OXF.BRC.1, the patients are divided into four classes: LumA, LumB, Her2 and Basal. We observed eight relevant clusters, four of which are subclasses of class LumA (cluster 4 - pvalue 2.50 ×10^−4^; cluster 5 - pvalue 8.71 ×10^−8^; cluster 6 - pvalue 2:92 ×10^−3^; cluster 11 - pvalue 1.97 ×10^−3^) and two are subclasses of class LumB (cluster 2 - pvalue 3:93 ×10^−14^; cluster 10 - pvalue 5:14 ×10^−3^). We also report the influence of each data on the final cluster. While it is obvious that the clusters are obtained considering all the genomic data views, the information needed to identify a specific subclass can be more relevant in a particular data type instead over the others. For example, the clusters 3, 6 and 11 of the OXF.BRC.1 dataset are both labeled as LumA. miRNA expression contributes for the 100 % to define the cluster 11, the gene expression is mainly determining the cluster 3 (57 %), while for cluster 6 they are equally important. This could mean, for example, that patients in cluster 11 are particularly characterized by miRNA expression while patients in cluster 3 by gene expression.

**Fig. 3:**
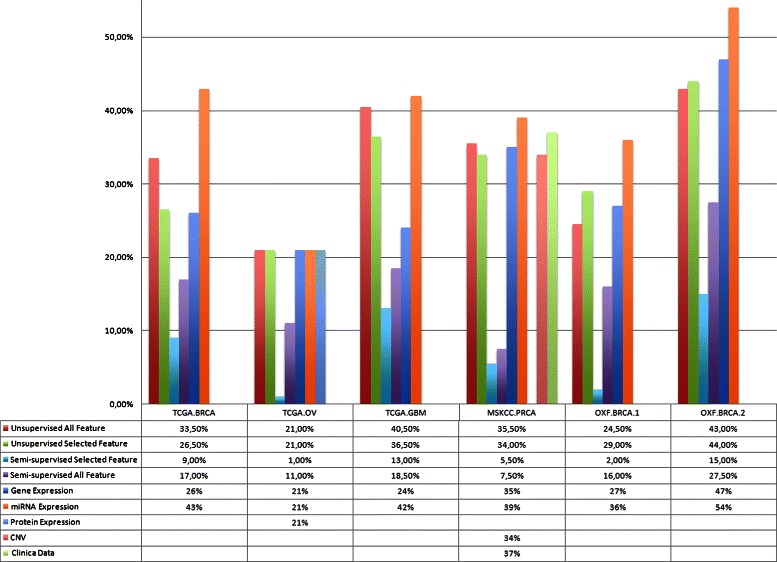
Cluster Impurity difference between single view and integration analysis: Cluster impurity was evaluated as the fraction of objects that were inconsistent with the label of the cluster. It was calculated using each data type alone and by integrating them. Errors decreased with the integration approach in particular when the semi-supervised methodologies were used

As shown in Fig. 3, the integrative clustering performed generally better that the clustering on each single data view. In the TCGA.BRCA dataset, the mean cluster impurity is about 26 % when patients are grouped by the gene expression and 43 % when they are grouped by their miRNA expression profiles. However, combining the gene and the miRNA expression profiles, 26,50 % of error in unsupervised mode and 9 % in semi-supervised mode are obtained, respectively. Only in a few cases, the patient grouping based on a single data view performs better than the one obtained with multiple data types.

**Fig. 4:**
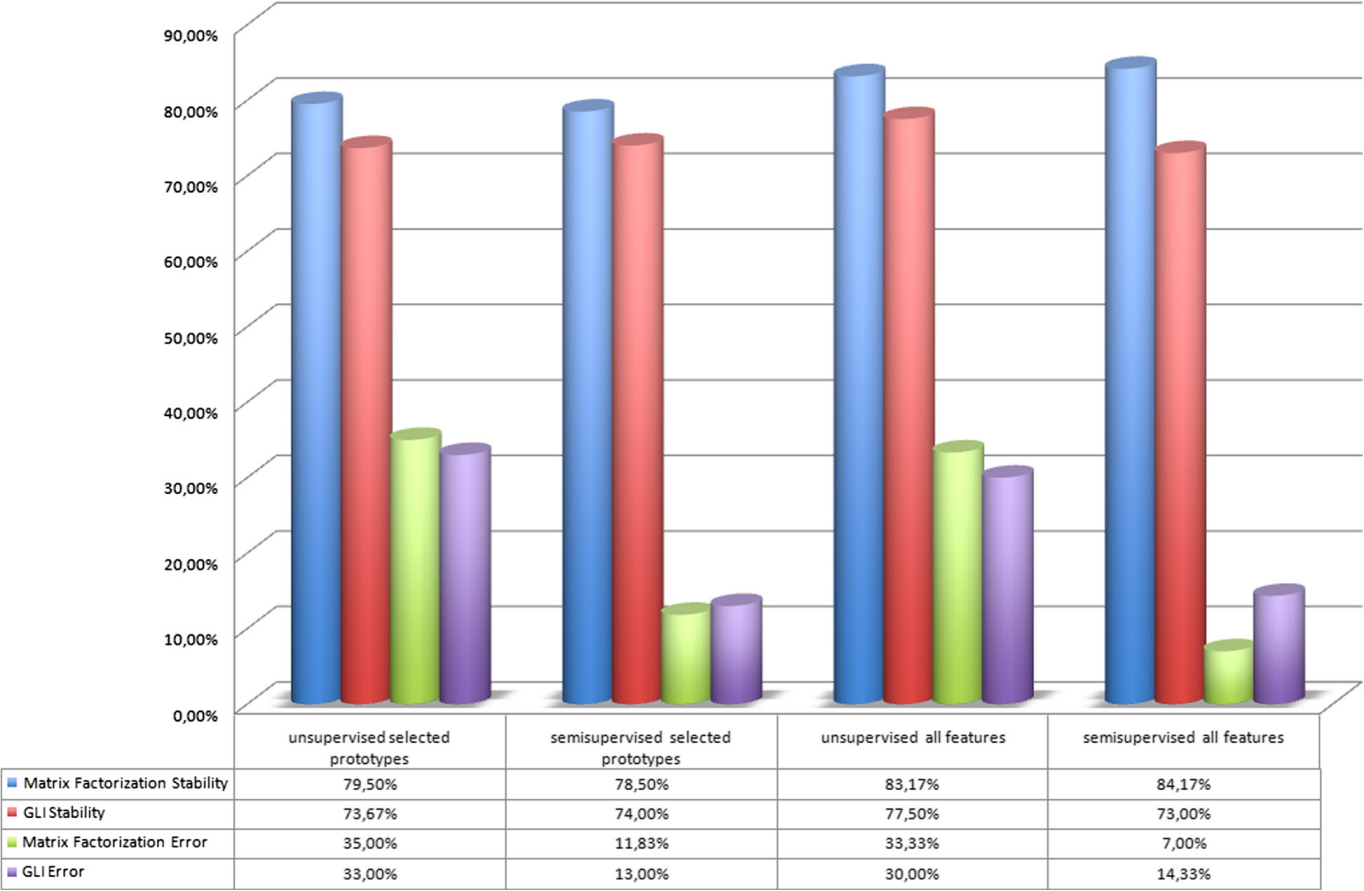
Difference between alternative integration methods: The mean cluster stability is reported, as calculated on four covariates represented by the type of experiment executed. Clustering stability was calculated by comparing the unsupervised and the semi-supervised mode, both using either all the features or only the selected prototypes

Figure 4 depicts the comparison between the two integration methods, either with or without prior information. The matrix factorization based method reaches the higher stability (about 85 %) in all the cases. With respect to the cluster impurity, the difference is almost always negligible. The greatest difference occurs when passing from the unsupervised to the semi-supervised approach. The cluster impurity for the unsupervised clustering is about 30 % and about 7 % for semi-supervised. Therefore, for more accurate sub-typing of classes semi-supervised integration was used, which maintains high stability and reduces the classification error compared to the classes. However, in case of unbalanced patient classes, the prior information is needed to increase the prediction.

Since we tested different algorithms at each step of our methodology, we aimed at understanding if a common pipeline for all the datasets could be applied. After the execution of all the analyses, we observed that the best algorithms for the first and second steps strongly depend on the data. We found that K-means is the best algorithm for step 3 for the TCGA.BRACA, OXF.BRCA.1 and OXF.BRCA.2 datasets (Table 2). At the last step, the matrix factorization approach provided lower errors and greater stability as compared with the general linear integration methods on the majority of the datasets. This result corroborates our hypothesis that a late integration approach is better for it allows using the best algorithms for each data type.

**Table 2:** Best combination of methods for each step: Summary of the best combination of algorithms for each view used to obtain the best grouping of patients that identifies significant sub-classes

		(a)	(b)	(c)	(d)
Dataset	Views	Feature	Feature	Patients	Late
		clustering	selection	clustering	integration
TCGA.BRCA	RNASeq	Pam	CAT-score	Kmeans	MF
	miRNASeq	Pam	CAT-score	Pam	
TCGA.OV	Gene Expression	Pam	Random Forest	DM	MF
	Protein Expression	Pam	-	DM	
	miRNA Expression	Pam	-	DM	
TCGA.GBM	Gene Expressions	Spectral	CAT-score	Kmeans	MF
	miRNA Expression	Ward	-	Kmeans	
OXF.BRCA.1	Gene Expressions	Pam	Random Forest	Ward	GLI
	miRNA Expression	Pam	Random Forest	Kmeans	
OXF.BRCA.2	Gene Expressions	Pvcluster	CAT-score	Kmeans	MF
	miRNA Expressions	Pam	Random Forest	Kmeans	
MSKCC	Gene Expressions	Pam	CAT-score	Kmeans	MF
	miRNA Expressions	Pam	-	Pam	
	CNV	Spectral	CAT-score	Kmeans	
	Clinical	-	-	Pam	

In the feature selection column the symbol (-) means that feature selection was not executed because the number of features was small. Symbol (DM) in Patient clustering column means that same classification error was obtained with all the algorithms used

In order to evaluate the performance of the proposed method, we systematically compared it with Tw-Kmeans and SNF algorithms (Table 3). Anyhow, we did not compare our method with iClust, as it has been show to have worse performance than SNF, with which we deal in this study [9]. We confirmed that late integration works more efficiently in integrating different views of genomic data. This is due to the large complexity and difference between the views. When views have different numerical and statistical characterizations, it is more convenient to individually analyze single data types and then combine the results in a multi-view analysis. This becomes more and more important as the number of views involved in the analysis increases.

**Table 3:** Validation Results: The mean classification error, normalized mutual information (NMI) and stability, on all datasets, are shown, measuring the agreement between the clusters resulting from an approach and the real patient classification

	Feature	Integration	Algorithm	Error	NMI	Stability
Single View	All Feature	-	Ward	30,08 %	26 %	86 %
		-	Kmeans	30,93 %	25 %	51 %
		-	Pamk	30,75 %	24 %	94 %
	Selected Prototype	-	Ward	30,72 %	26 %	89 %
		-	Kmeans	30,36 %	25 %	52 %
		-	Pamk	30,78 %	24 %	96 %
Multi-View	All Feature	Early Integration	Tw-kmeans	37,10 %	24 %	69 %
	All Feature	Intermediate Integration	SNF	30,83 %	22 %	83 %
	All Feature in Cluster of Selected Prototype	Intermediate Integration	SNF	31,31 %	18 %	82 %
	Selected Prototype	Late Integration unsupervised	MF/GLI	**27,47 %**	**28 %**	**85 %**
	Selected Prototype	Late Integration semi-supervised	MF/GLI	**6,30 %**	**63 %**	**84 %**

Bold font in percentage indicates best performance in the experiments

## Evaluation of genes in breast cancer datasets

**Fig. 5:**
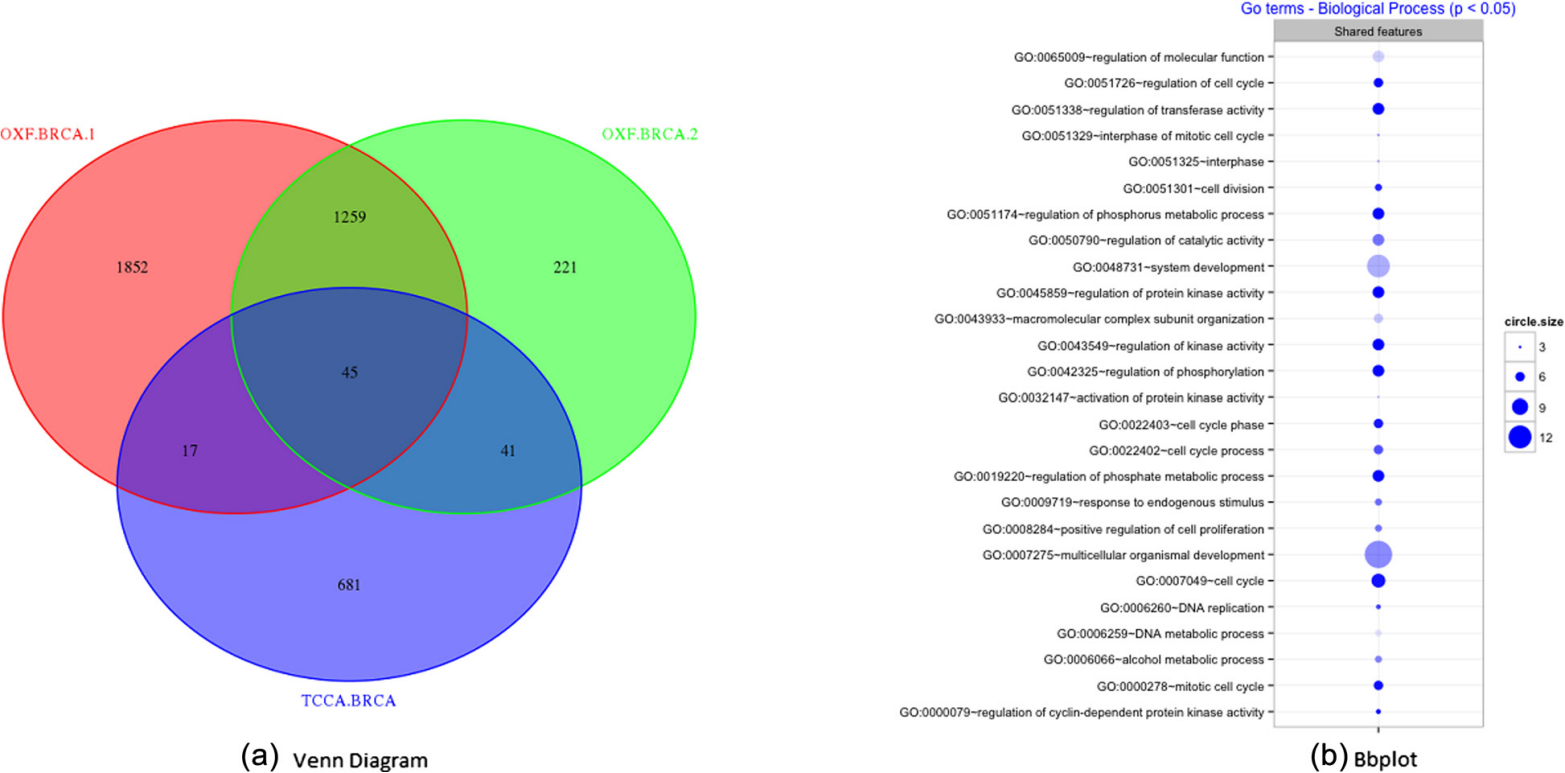
Breast Cancer Gene Analysis: (**a**) the Venn diagram shows the number of common relevant genes between the three datasets. The analysis highlights 45 common genes between the three lists. (**b**) The bubble plot displays the enriched GO terms found by using DAVID. A transparent bubble indicates a set of significant genes, a dark bubble indicates a set of highly significant genes. The diameter of the bubble indicates the number of genes related to the same GO term

We selected a robust set of features from each analyzed dataset in order to find common features (Fig. 5 a) and highlight shared patterns by enrichment analysis (Fig. 5 b). Each list of features was obtained by using the Borda-count rule across the leave-one-out replicates. The enrichment analysis was performed by using the DAVID functional annotation tool [18, 19] and graphically displayed with the R package BACA [20]. Figure 5 b reports a chart indicating unique and common Gene Ontology (GO) terms found by using DAVID on the different lists. It is possible observe that the three lists of features highlight similar GO annotations, involved for instance in regulation of kinase activity and regulation of cellcycle. The list of genes shared between the three breast cancer datasets can be found in Additional file 3.

## Conclusions

In this study, we proposed a methodology for multiple genomic data type analysis aiming at patients subtyping. The methodology is composed of four steps using state of the art algorithms. Furthermore we systematically searched for the best algorithm for each step on six of benchmark datasets. We performed experiments in a late integration fashion, with two different algorithms. Since we were interested in high accuracy in class patient subtyping, we used prior information as a new view in the integration process. We found that the integrative clustering outperforms the single view approaches on all the datasets. We also showed that our method is stable by executing clustering on perturbed datasets removing one patient at a time and evaluating the normalized mutual information between all the resulting clusterings.

## Methods

The proposed methodology for the analysis of multi-view biological datasets takes in input *n* matrices $M_{i} \in R^{F_{i} \times P}\; for \;i=1, \ldots, n \phantom {\dot {i}\!}$, where *F*_*i*_ is the number of features (genes, miRNAs, CNV, methylation, clinical information, etc.) and *P* is the number of patients and a vector *cl* of classes labels, and yields a multi-view partitioning $G = \bigcup _{i=1}^{k}(G_{i})$ of patients. The multi-view integration methods also return a matrix *C* where *c*[ *i*,*j*] is the contribution of view *i* to the final multi-view cluster *j*.

The approach consists of four main steps as shown in Fig. 1:

1. Prototype Extraction: for each view, the features were filtered by variance and clustered in order to find prototypes, reducing the input data dimension.
2. Prototype ranking: the prototypes found in the step 1 were ranked based on their ability to separate the classes.
3. Single view clustering: in each view, the samples were clustered using the prototypes created in the steps 1 and 2 as features
4. Integration: single view clustering results were integrated with a late integration approach, in order to obtain the k final multi-view meta-clusters

The late integration methodology can be considered as a further step of the proposed data mining pipeline, in which the clustering results of each single view are unified. This approach offers a number of significant advantages: (i) clustering algorithms can be optimally chosen with respect to each single view; (ii) it can be naturally parallelized; (iii) representation issues are avoided since clustering results are the inputs to the integration algorithms.

### Prototype extraction

The features with low variance across the samples were eliminated. Therefore the data were clustered with respect to the patients and the cluster centroids were selected as the prototype patterns. The centroid of each cluster was selected as the most correlated element with respect to the other elements in that cluster. Different clustering algorithms were used: Pvclust [21], SOM [22], hierarchical clustering with Ward’s method [23], K-means [24], Partitional Around Medoids [25] and Spectral clustering [26].

The idea is to evaluate several popular clustering techniques and compare their behaviour on the different views with respect to the hierarchical method that is the standard algorithm used to cluster genes. As noted in [27], cluster analysis is a complex and interactive process and results change based on its parameters. Therefore, each algorithm was executed for different values of K. For each algorithm and for each K, clustering performance was evaluated according to the following evaluation function:

(1) $$\begin{array}{@{}rcl@{}}  VAL = \frac{1}{4}\left(\frac{IC+1}{2} + 1-\frac{EC+1}{2} + (1-S) + CG\right) \end{array} $$

where *IC* is the complete diameter measure, representing the average sample correlation of the less similar objects in the same cluster; *EC* is the complete linkage measure, representing the average sample correlation of the less similar objects for each pair of clusters; *S* is the singleton factor and *CG* is the compression gain. The evaluation function was defined in order to obtain the output value normalized between 0 and 1. The complete diameter and the complete linkage measures were calculated with the R “clv” package [28]. The number of singleton was normalized in a range (0,1) in order to be comparable with the correlation measure. It was defined as *S*=*N*/(*K*−1). The compression gain was defined as *C* *G*=1−(*K*/*N*_*elem*_), where *K* is the number of clusters and *N* is the number of elements to be clustered.

Each clustering algorithm was executed on n different values of K and the corresponding results were evaluated with the function VAL. Values close to 1 indicate a clustering with similar objects in the clusters, weakly linked clusters, with few singletons and with a good compression rate. A numeric score was then assigned to each K value by considering the average values of the VAL function compiled over the clustering results obtained with the different algorithms. Then, the K showing the highest score was chosen and subsequently used to identify the best clustering algorithms having the first two highest scores with respect to the selected k value. In Algorithm 1 is reported the computational procedure followed to fine-tuned the k-values for the cluster analysis.



### Feature ranking

If the number of prototypes, after the fist step, was still high, further dimensional reduction by feature selection was done. Feature ranking was performed by computing the CAT-score [29] and the Mean Decreasing Accuracy index calculated by Random Forests [30]. The parameters of RF-based classifiers were fine-tuned by using the R package rminer [31]. It provides a function that first tunes the hyper parameter(s) of a selected model by using bootstrap methods and subsequently builds the corresponding supervised data-mining model. For each rank, the cumulative sum of the ranking score was computed and four different cuts based on the cumulative values were taken. Cuts took into account all the features needed to maintain 60 %, 70 %, 80 % and 90 % of the cumulative value. An example is shown in section Prototype Extraction of Additional file 1. These different groups of features were used to cluster patients in each single view, with the same single view clustering algorithms used in the previous step. The number of clusters *K* was considered as the number of classes. For each clustering, the error was calculated as the dispersion obtained in the confusion matrix between class labels and clustering assignments. The clustering algorithm that reached the minimum error for each view was then selected. These clustering results were used as the input to the late integration step.

### Integration

Two late integration methods were used: the matrix factorization approach [11] and a general model for multi-view integration [10]. The first method [11] combines information by factorizing the membership matrix of patient single-view clusterings. The method starts by transposing all the membership matrices and stacking them vertically obtaining the matrix of cluster *X*∈*R* ^*lXn*^ where *l* is the total number of cluster in *C*. The objective is to find the best approximation of *X* such that

(2) $$ X = PH \; and \; P >= 0, H >= 0  $$

The results of the factorization are two matrices: *P*∈*R* ^*lXk*^ that projects the clusters in a new set of *k* meta-clusters and *H*∈*R* ^*kXn*^ whose columns can be viewed as the membership of the original objects in the new set of meta-clusters. Based on the values in the projection matrix *P*, we can calculate a matrix *T*∈*R* ^*vXk*^. *T*_*hf*_ indicates the contribution of the view *V*_*h*_ to the f-th meta-cluster. Based on values in *P* it is also possible to find the optimal value of *k* for the number of multi-view clusters we want in output. The matrix factorization was run with a range of values for *k* as input and the algorithm returns the factorization for the best value of *k*.

The second method exploits the intuition that the optimal clustering is the consensus clustering shared by as many views as possible. This can be reformulated as an optimization problem where the optimal clustering is the closest to all the single view clusterings under a certain distance or dissimilarity measure. Clusterings are again represented as membership matrices.

Formally the model can be described as follow: given a set of clustering membership matrices $M=\;[\!M_{1},\ldots, M_{h}] \in R_{+}^{n \times l}$ and a positive integer *k*, the optimal clustering membership matrix $B \in R_{+}^{n \times k}$ and the optimal mapping matrices $P = \,[\!P_{1},\ldots,P_{h}] \in R_{+}^{k \times l}$ are given by the minimization:

(3) $$ \begin{aligned} & \underset{B,P}{\text{min}} & & GI(M||BP) \\ & \text{s. t.} & & P \geq 0\\ & & & B \geq 0, B\mathbf{1}=\mathbf{1} \end{aligned}  $$

where *G* *I*(*M*||*B* *P*) is the generalized Kullback-Leibler divergence such that

$$GI(X||Y)=\sum_{ij}\left(logX_{ij} log \frac{X_{ij}}{Y_{ij}} - X_{ij} + Y_{ij}\right) $$

subject to the constraint that both *P* and *B* must be non-negative and that each row of *B* must sum to one.

By taking the membership matrix for each of the previous clusterings, and, using these two late integration methods, a multi-view clustering was obtained. Experiments were performed in two ways: the former uses all the prototypes for classification; the latter uses only the most relevant ones for class separability. Each one of these approaches were performed both in unsupervised and semi-supervised manners, respectively.

The semi-supervised approach consists of giving a priori information as input to the techniques of late integration via a membership matrix of patients with the exact information of their classes. This information is combined with the membership of the patients compared to the single view clustering and integrated in metaclusters. This can be a useful approach mainly when the data set is composed of unbalanced or under represented classes.

### Derivation of subclasses

Once the multi-view clusters were obtained, a subclass was assigned to each one. For each cluster, the number of objects of each class was calculated and the class with more representative patterns was assigned as the cluster label. Then, a p-value was calculated in order to verify the statistical significance of the subclass by the Fisher’s exact test [32].

### Validation

The method was compared with classical single view clustering algorithms, early and intermediate integration approaches. For each method clustering impurity, normalized mutual information (NMI) and cluster stability were evaluated. Cluster impurity was defined as the number of patients in the cluster whose label differs from that of the cluster. Given two clustering solutions *C* *l*_1_ and *C* *l*_2_ NMI was computed as the mutual information between the two clustering normalized by the cluster entropies. The NMI was computed between clustering results and real patient classifications.

Since prior information was introduced, the stability of the system was tested with leave-one-out technique. A test in itself was run on the first step to generate a stability index for the prototypes of the obtained clusters. Then, the steps 2, 3 and 4 were evaluated jointly to assess the stability of the selected features and to evaluate the robustness of the multi-view clustering results. Furthermore, a borda-count [33] method was performed to find the final list of features selected over the leave-one-out experiments for the integration step.

At the end of this process, N different clustering assignments were obtained, one for each removed patient. An *N*×*N* matrix M was created, where *M*(*i*,*j*) was the normalized mutual information (NMI) between the clustering obtained removing patient i and the clustering obtained removing patient *j*. Then the mean of the matrix M was calculated, indicating the stability measure of the method.

The comparison study involved the following methods:

- Kmeans, Hierarchical and Pam single view clustering
- Tw-Kmeans, an early integration multi-view clustering algorithm
- SNF, an intermediate integration multi-view clustering algorithm

Experiments with single view clustering algorithms were executed in feature concatenation mode: data from views were concatenated and used as a new greater feature space. This kind of experiments were run both on the most variable features for each view and on the most relevant prototypes found after the first and second steps of our approach. Experiments with Tw-kmeans were executed on all the features without any manipulation of the initial datasets. Experiments with SNF were executed both using all the features and using all the features that belong to the clusters associated to the relevant prototypes.

### Dataset collection and preparation

Six datasets were downloaded from The Cancer Genome Atlas (TCGA) (https://tcga-data.nci.nih.gov/tcga/), Memoral Sloan-Kettering Cancer Center (http://cbio.mskcc.org/) and from NCBI GEO (http://www.ncbi.nlm.nih.gov/geo/) (See Table 1).

#### TCGA.BRC

Breast cancer dataset from the TCGA repository (https://tcga-data.nci.nih.gov/tcga/ - Breast invasive carcinoma [BRCA]). The samples in this dataset correspond to breast cancer patients with invasive tumors. Genomic data for two views were downloaded: RNASeq and miRNASeq (Level 3). Because level 3 data corresponds to already preprocessed data, only the batch effect was removed by the comBat method in the R “sva” package [34]. Patients were subsequently divided into four classes (Her2, Basal, LumA, LumB), using PAM50 classifier [35, 36].

#### OXF.BRC.1

Breast cancer dataset from a study performed at Oxford University [37]. Data were downloaded from Gene Expression Omnibus Dataset (http://www.ncbi.nlm.nih.gov/geo/). Data were available for two views: mRNA and microRNA expression under the accession number GSE22219 and GSE22220. Patients were divided into four classes (Her2, Basal, LumA, LumB), using PAM50 classifier [35, 36].

#### OXF.BRC.2

Breast cancer dataset from a study performed at Oxford University [37]. Data were downloaded from Gene Expression Omnibus Dataset (http://www.ncbi.nlm.nih.gov/geo/). Data were available for two views: mRNA and microRNA expression under the accession number GSE22219 and GSE22220. Patients were divided into four classes (Level1, Level2, Level3, Level4) using clinical data also retrieved from the same source. See Table 4 for classes definition.

**Table 4:** Oxford Dataset: Oxford Dataset, class definition by clinical data

Class	Clinical information
Level1	er = 1, node = 0, grade = 1–2
	er = 1, node = 0, grade = 3–4
Level2	er = 1, node > 0, grade = 1–2
	er = 1, node > 0, grade = 3–4
Level3	er = 0, node = 0, grade = 1–2
	er = 0, node = 0, grade = 3–4
Level4	er = 0, node > 0, grade = 1–2
	er = 0, node > 0, grade = 3–4

#### TCGA.GBM

Glioblastoma cancer dataset from the TCGA repository. The samples in this dataset correspond to glioblastoma patient with invasive tumors. TCGA website was accessed (https://tcga-data.nci.nih.gov/tcga/ - Glioblastoma multiforme [GBM]) and publicly available data for two views were downloaded: gene expression and miRNA expression. Also clinical data was retrieved. The patients were divided info four classes: Classical, Mesechymal, Neural and Proneural as described in [38].

#### TCGA.OVG

Ovarian cancer dataset from the TCGA repository (https://tcga-data.nci.nih.gov/tcga/ - Ovarian serous cystadenocarcinoma [OV]). The samples in this dataset correspond to patient affected by ovarian serous cystadenocarcinoma tumors. Publicly available data for three views were downloaded: gene expression, protein expression, and miRNA expression. Clinical data were downloaded in order to classify patients in three categories. In particular patients were classified by clinical stage: first class: stage IA, IB, IC, IIA, IIB and IIC, second class: IIIA, IIIB and IIIC, third class Stage IV.

#### MSKCC.PRCA

Prostate cancer dataset from a study performed at the Memorial Sloan Kettering Cancer Center (http://cbio.mskcc.org/). The samples in these datasets correspond to patient prostate cancer tumors. The MSKCC Cancer Genomics data portal (http://cbio.mskcc.org/cancergenomics/prostate/data/) was accessed and data for five views were downloaded: clinical data, gene expression, microRNA expression and copy number variation. Patients were classified in two classes by using clinical data by the tumor stage: class one is Tumor Stage I and class two is Tumor Stage II, III and IV. Classification of patient was done according to a previous study performed on the same dataset [14].

## Additional files

Additional file 1
**It contains a section for each step of the methodology in which the tables and figures with the results for each dataset are reported.** (PDF 1495 kb)

Additional file 2
**Each sheet refers to each dataset analysed, reporting the results of the single-view clustering patients.** Clustering errors for each algorithm and each cut of feature are also reported. (XLSX 54 kb)

Additional file 3
**It contains the gene symbols and description for all shared genes between the tree breast cancer datasets highlighted by the analysis.** (DOCX 14 kb)

## Abbreviations

miRNA: MicroRNA
CNV: Copy number variation
NMI: Normalized mutual information
GO: Gene Ontology
SOM: Self-organizing map
Pam: Partitional around medoids
CAT-score: Correlation-adjusted t-score
DAVID: Database for annotation, visualization and integrated discovery
TCGA: The Cancer Genome Atlas
MSKCC: Memorial Sloan Kettering Cancer Center
GEO: Gene Expression Omnibus
