# Supplementary material for: MVDA: a multi-view genomic data integration methodology
Source: BMC Bioinformatics. 2015 Aug 19;16:261. doi: 10.1186/s12859-015-0680-3 (PMC4539887; doi:10.1186/s12859-015-0680-3)
Supplement: Additional file 1 — It contains a section for each step of the methodology in which the tables and figures with the results for each dataset are reported. (PDF 1495 kb) [file 12859_2015_680_MOESM1_ESM.pdf]

MVDA: A multi-view genomic data integration methodology: Supplementary Materials

A. Serra, M. Fratello, V. Fortino, G. Raiconi, R. Tagliaferri and D. Greco

Prototype Extraction

In this section intermediate results of step one of the methodology is reported. As a preliminary step, for all dataset, feature with low variance are eliminated. Variance was evaluated for each feature and then the cumulative function of the variance was calculated. The the cumulative function was cut at different level as showed in an example in figure 1.

Then feature were clustered by correlation in order to remove feature redundancy and reduce their number.

Here are reported evaluation metrics for each algorithm in clustering feature as described in section material and method of the manuscript. For each dataset the best two algorithms that reach higher value of the metric were selected.

In figures 2 3 4 5 6 we can see the algorithms behavior by varying the value of K. More details are shown in table 1.

Figure 1 Feature ranking cut: here IS reported, as example, the feature ranking cut for the gene expression view of the OXF.BRC.1 dataset. Feature ranking was performed with the Cat-t score method on the prototypes obtained with the Pam algorithm. As we can see, in order to achieve the 60% of the cumulative ranking score 53 prototypes were needed, 71 for 70%, 93 for 80% and 126 on 90%. In this example there were 200 prototypes at all.

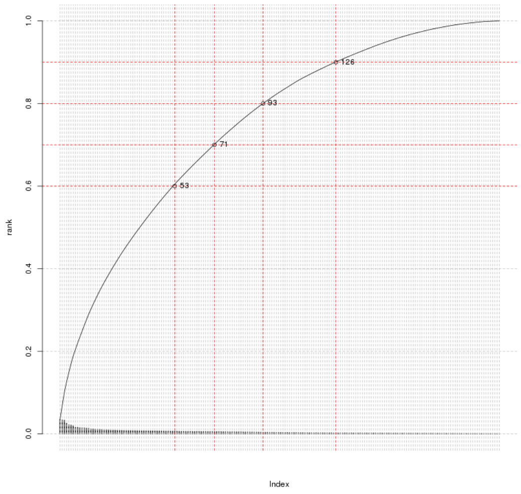

Table 1 Results after step 1: Here we show the results of the two best algorithms used in order to cluster elements in each view for each dataset. For each dataset the top 20% of features were selected. N is the number of patients in each dataset. Apart for Pvclust algorithm that automatically finds the number of clusters, the optimal value of K was calculated as described in section Material and Methods (the optimal values are those in the red lines). For each dataset the two best algorithms that maximize the index (bold value) were selected

View	Feature	Variable Feature		SOM	Pam	K-means	Ward	Spectral	Pvclust	
**Breast cancer patient samples from The Cancer Genome Atlas (TCGA), N = 151**										
			Prototype			Index			Prototype	Index
RNASeq	20510	4100	100	0,82	0,84	0,77	0,83	0,82	**297**	**0,86**
			**200**	0,85	**0,87**	0,82	0,85	0,85		
			300	0,75	0,85	0,78	0,84	0,83		
			400	0,75	0,82	0,80	0,82	0,82		
			500	0,70	0,78	0,78	0,83	0,82		
miRNASeq	1046	209	5	0,80	0,80	0,79	0,80	0,80	**24**	**0,84**
			10	0,80	0,82	0,78	0,82	0,80		
			**20**	0,81	**0,84**	0,77	0,83	0,83		
			30	0,64	0,72	0,73	0,80	0,79		
			40	0,60	0,68	0,68	0,78	0,78		
**OXF.BRC.1 and OXF.BRC.2 breast cancer patient samples from the Gene Expression Omnibus (GEO), N = 201**										
			Prototype			Index			Prototype	Index
Gene Expression	21439	4288	100	0,80	0,82	0,76	0,79	0,78	**237**	**0,85**
			**200**	0,83	**0,86**	0,81	0,83	0,83		
			300	0,74	0,84	0,78	0,82	0,82		
			400	0,74	0,78	0,76	0,82	0,81		
			500	0,71	0,74	0,75	0,82	0,82		
miRNA Expression	734	147	5	0,76	0,82	0,76	0,82	0,81	**30**	**0,84**
			10	0,76	0,83	0,73	0,82	0,82		
			**20**	0,81	**0,84**	0,80	0,83	0,83		
			30	0,64	0,74	0,72	0,78	0,76		
			40	0,62	0,68	0,69	0,76	0,75		
**MSK.PRCA prostate cancer patient samples from Memorial Sloan-Kettering Cancer Center (MSKCC), N=88**										
			Prototype			Index			Prototype	Index
Gene Expression	26446	5200	**100**	0,86	**0,89**	0,87	0,88	**0,89**	532	0,85
			200	0,84	0,88	0,87	0,88	0,89		
			300	0,83	0,87	0,83	0,86	0,87		
			400	0,76	0,84	0,82	0,86	0,86		
			500	0,71	0,81	0,80	0,85	0,86		
miRNA Expression	368	75	**5**	0,83	**0,84**	0,83	**0,85**	0,84	2	0,81
			10	0,74	0,82	0,80	0,81	0,82		
			15	0,67	0,79	0,72	0,79	0,80		
			20	0,57	0,70	0,69	0,80	0,79		
Copy Number	18000	3600	**100**	0,85	**0,86**	0,85	0,86	**0,87**	258	0,84
			200	0,85	0,85	0,85	0,85	0,85		
			300	0,84	0,80	0,80	0,84	0,84		
			400	0,83	0,79	0,78	0,82	0,83		
			500	0,81	0,78	0,76	0,82	0,83		
Clinical	9	-	-	-					-	-
**TCGA.GBM glioblastoma multiform samples from The Cancer Genome Atlas (TCGA), N = 167**										
			Prototype			Index			Prototype	Index
Gene Expression	12042	2408	**50**	0,85	**0,87**	0,82	0,86	**0,87**	306	0,86
			100	0,79	0,86	0,79	0,85	0,86		
			150	0,67	0,83	0,79	0,84	0,85		
			200	0,65	0,79	0,77	0,84	0,84		
			250	0,62	0,80	0,79	0,83	0,84		
miRNA Expression	534	107	5	0,84	0,84	**0,85**	**0,85**	0,84	2	0,79
			10	0,83	0,84	0,78	0,84	0,84		
			15	0,67	0,83	0,76	0,83	0,83		
			20	0,63	0,80	0,74	0,81	0,81		
**TCGA.OVG ovarian cancer patient samples from The Cancer Genome Atlas (TCGA), N=93**										
			Prototype			Index			Prototype	Index
Protein Expression	166	-	5	0,82	0,83	0,77	0,82	0,82	32	0,79
			10	0,82	**0,83**	0,78	**0,84**	0,82		
			15	0,73	0,83	0,76	0,83	0,72		
			25	0,66	0,82	0,77	0,82	0,81		
miRNA Expression	800	201	5	0,84	0,84	0,84	**0,85**	0,84	31	0,81
			10	0,85	0,84	0,77	0,85	0,85		
			15	0,77	0,84	0,81	0,85	0,85		
			20	0,68	0,84	0,79	0,84	0,85		
			25	0,64	0,83	0,77	0,83	0,83		
Gene Expression	12043	3011	50	0,84	**0,85**	0,81	**0,85**	0,84	423	0,81
			100	0,82	0,85	0,77	0,85	0,85		
			200	0,74	0,83	0,75	0,83	0,83		
			300	0,65	0,82	0,73	0,82	0,82		
			400	0,63	0,78	0,71	0,81	0,81		
Clinical	25	-	-	-					-	-

Figure 2 MSKCC step1 figure

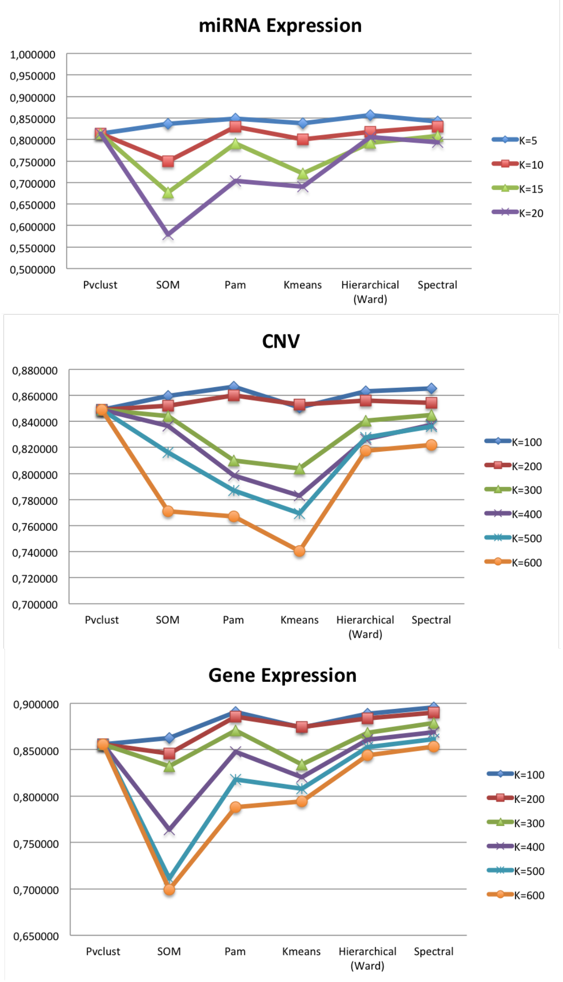

Figure 3 TCGA.GBM step1 figure

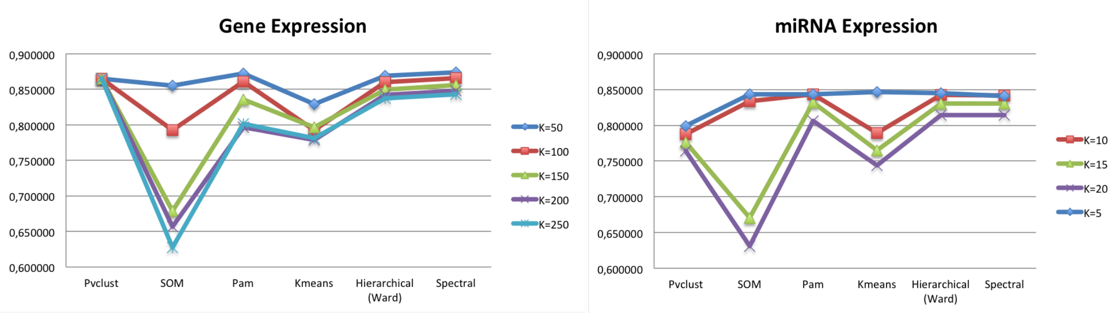

Figure 4 TCGA.BRCA step1 figure

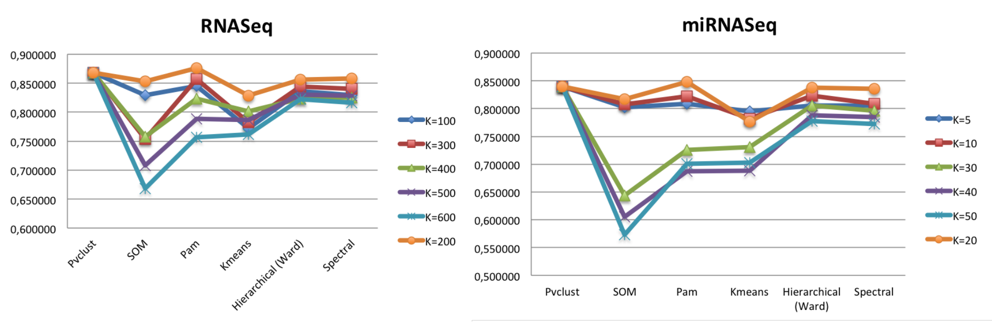

Figure 5 TCGA.OVG step1 figure

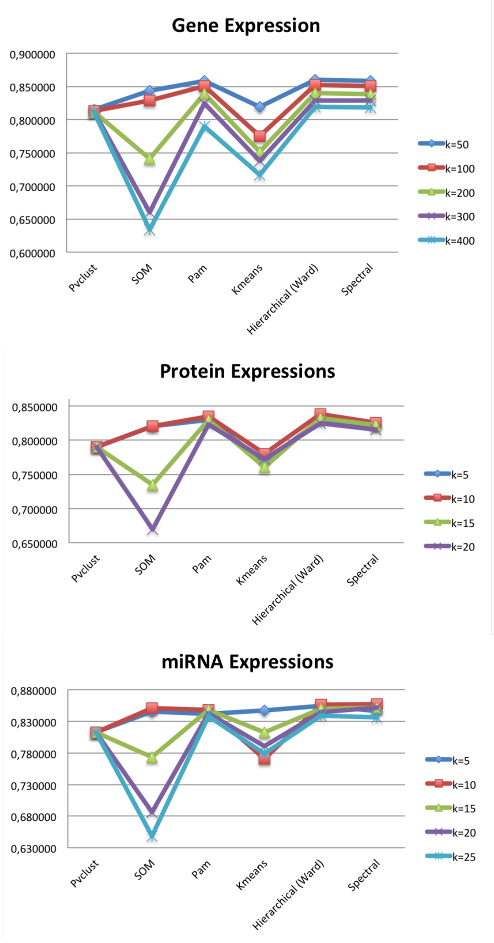

Figure 6 OXF.BRCA.1 and OXF.BRCA.2 step1 figure

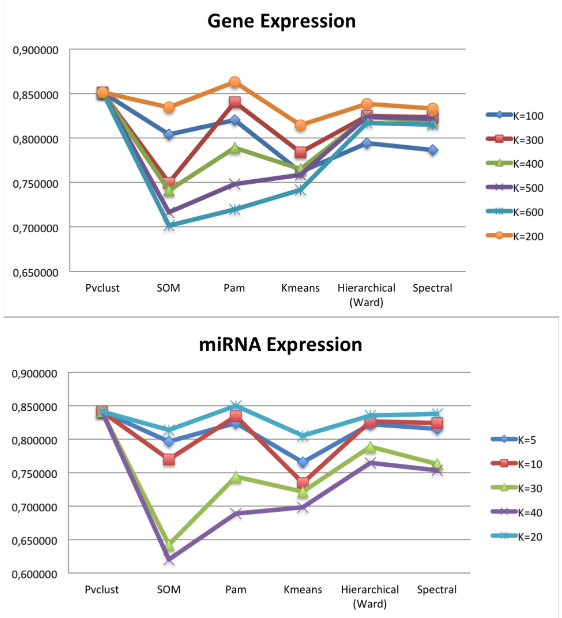

Single view patients clustering

In this section summary results of single view patient clustering for each dataset are showed. The clustering algorithm reach the minimum impurity error percentage is also reported. Table 2 reports which cut is used in order to reach this results and also the algorithm (used in the first step of the methodology) from witch the prototype come from.

Table 2 Single view clustering results after the feature selection step

Dataset	View	Single View algorithm	Ranking	Cut	Prototype from	N.Cluster	Error
**Breast Cancer patients from The Cancer genome Atlas (TCGA), N = 151**							
TCGA.BRC	RNASeq	K-means	Cat-t Score	60%	Pamk	4	0.17
	miRNASeq	K-means	Cat-t Score	80%	Pamk	4	0.37
**Breast Cancer patients Samples from The Gene Expression Omnibus (GEO), N = 201**							
OXF.BRC.1	Gene Expression	K-means	Random Forest	70%	Pamk	4	0.17
	miRNA Expression	K-means	Random Forest	90%	Pvclust	4	0.32
**Breast Cancer patients Samples from The Gene Expression Omnibus (GEO), N = 201**							
OXF.BRC.2	Gene Expression	K-means	Cat-t score	70%	Pvclust	4	0.47
	miRNA Expression	K-means	Random Forest	90%	Pamk	4	0.54
**Breast Cancer patients from The Cancer genome Atlas (TCGA), N = 151**							
MSKCC.PRA	Gene Expression	K-means	Cat-t score	80%	Pamk	2	0.31
	miRNA Expression	Pam	-	-	Pamk	2	0.39
	Copy Number	Ward	Random Forest	90%	Spectral	2	0.31
	Clinical	Pam	-	-	-	2	0.37
**Glioblastoma Multiforme patients from The Cancer genome Atlas (TCGA), N = 167**							
TCGA.GBM	Gene Expression	K-means	Cat-t score	90%	Spectral	4	0.17
	miRNA Expression	K-means	-	-	Ward	4	0.42
**Glioblastoma Multiforme patients from The Cancer genome Atlas (TCGA), N = 398**							
TCGA.OVG	Gene Expression	K-means	Random Forest	-	Pamk	3	0.21
	miRNA Expression	K-means	-	-	Pamk	3	0.20
	Protein Expression	K-means	-	-	-	3	0.22

Final Results

In this section final results for all datasets are reported. All the results reported for the integration step refer to features obtained with the leave-one-out process. In particular table 3 shows cluster impurity errors and cluster stability computed for each dataset for the two integrative methods.

Relevant Prototype for each subclass

For each cluster of patients a set of features coming from different data types was available. Each cluster was analysed in order to find the features that characterize it better. Two kinds of analysis were performed: the former was the correlation between patients in the cluster, the latter was related to the distribution of each variable in one sample in a cluster compared to all the other samples. In the first case, the most relevant features for each cluster were identified by evaluating how the correlation between patients in one cluster decrease when a feature was removed. The feature relevance is directly related to the correlation decrease. One feature at a time was removed and the correlation was evaluated. At the end the features were ranked and the first features for each view were selected. Figure 8 shows the most relevant features for each dataset. In the second case, features were ranked for each cluster according to their distribution. The key concept was that the variance of a relevant feature is low in the cluster and high between clusters. So were considered significant those features for which the difference between the variance out of the cluster and the variance in the cluster were highest. The features were ordered according to this criterion and for each cluster was observed what are the top key features. An example of results on TCGA.BRCA dataset is reported in (Figure 7).

Class characterisation by visualisation

For inspection of the patient characteristics in each class, the distribution of each variable in a cluster was compared with its distribution in other clusters, using boxplots. A boxplot shows the median expression level (solid horizontal bar), the upper quartile and lower quartile range (shaded grey bar), the highest non-outlier and lowest non-outlier (smaller ticks joined by dashed lines), and any outlier (open circles). Because of the great amount of features the box-plot of all the variables cannot be visualized in a clear manner. So the features that gave more information on the difference between clusters were found. Analysis was started from cluster centroids. Feature were ranked by its variance between centroids. This means that the greater is the variance the greater is the difference between clusters for that feature. In (Figures 11), (Figure 12), (Figure 9) and (Figure 10) are reported the box-plot of each cluster calculated for these feature. Different behaviours in clusters related to different classes are clearly visible.

Figure 7 Variable Ranking according to the difference of variance inside and outside each cluster of the TCGA.BRCA dataset.

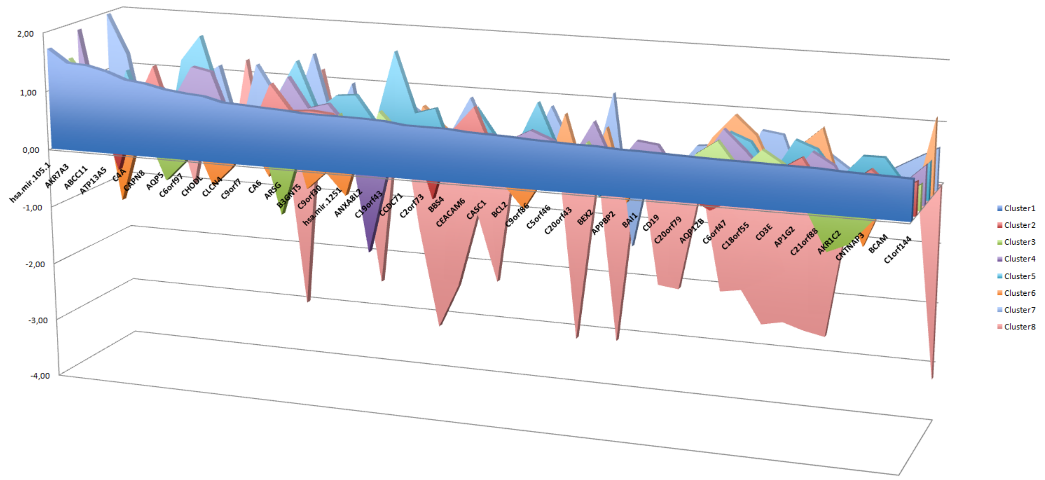

Table 3 Final results: the table shows the results for all the datasets for all the four experiments executed both with the matrix factorization approach and the general linear integration method.

**TCGA.BRC breast cancer patients from The Cancer Genome Atlas, N = 151**				
	Matrix Factorization		General Linear Integration	
	Error		Error	
	All Feature	Selected Feature	All Feature	Selected Feature
Semi supervised	12%	5,27%	13%	23%
Unsupervised	34%	26,64%	29%	27%
	Stability		Stability	
	All Feature	Selected Feature	All Feature	Selected Feature
Semi supervised	78%	77%	72%	73%
Unsupervised	76%	76%	63%	61%
**OXF.BRC.1 breast cancer patients from the Gene Expression Omnibus, N = 201**				
	Matrix Factorization		General Linear Integration	
	Error		Error	
	All Feature	Selected Feature	All Feature	Selected Feature
Semi supervised	9%	8%	23%	7%
Unsupervised	29%	26%	23%	29%
	Stability		Stability	
	All Feature	Selected Feature	All Feature	Selected Feature
Semi supervised	84%	70%	78%	69%
Unsupervised	84%	63%	75%	77%
**OXF.BRC.2 breast cancer patients from the Gene Expression Omnibus, N = 201**				
	Matrix Factorization		General Linear Integration	
	Error		Error	
	All Feature	Selected Feature	All Feature	Selected Feature
Semi supervised	25%	15,23%	16%	30%
Unsupervised	47%	33%	42%	34%
	Stability		Stability	
	All Feature	Selected Feature	All Feature	Selected Feature
Semi supervised	82%	77%	67%	71%
Unsupervised	63%	63%	75%	74%
**MSKCC.PRCA prostate cancer patients from Memorial Sloan-Kettering Cancer Center, N=88**				
	Matrix Factorization		General Linear Integration	
	Error		Error	
	All Feature	Selected Feature	All Feature	Selected Feature
Semi supervised	11%	1%	10%	5%
Unsupervised	36%	34%	33,20%	35%
	Stability		Stability	
	All Feature	Selected Feature	All Feature	Selected Feature
Semi supervised	85%	74%	73%	88%
Unsupervised	88%	72%	70%	73%
**TCGA.OVG ovarian cancer patient from The Cancer Genome Atlas, N = 398**				
	Matrix Factorization		General Linear Integration	
	Error		Error	
	All Feature	Selected Feature	All Feature	Selected Feature
Semi supervised	13%	1,5%	9%	2%
Unsupervised	20%	20%	21%	21%
	Stability		Stability	
	All Feature	Selected Feature	All Feature	Selected Feature
Semi supervised	88%	86%	80%	75%
Unsupervised	98%	97%	86%	86%
**TCGA.GBM glioblastoma multiform patients from The Cancer Genome Atlas, N = 167**				
	Matrix Factorization		General Linear Integration	
	Error		Error	
	All Feature	Selected Feature	All Feature	Selected Feature
Semi supervised	15%	7,78%	20%	12%
Unsupervised	36%	26%	40%	28%
	Stability		Stability	
	All Feature	Selected Feature	All Feature	Selected Feature
Semi supervised	88%	87%	77%	77%
Unsupervised	90%	90%	76%	73%

Figure 8 Feature relevance: for each multi-view clustering the most relevant features was identified in each cluster by evaluating the correlation reduction when a feature was removed. More relevant features are related to a greater decrease of the correlation reduction. Here are reported results only for the semi-supervised experiments that involve ranked prototypes. The x-axis reports the number of clusters while the y-axis reports the feature relevance. The feature relevance index goes from 1 (highly relevant) to 5 (not relevant).

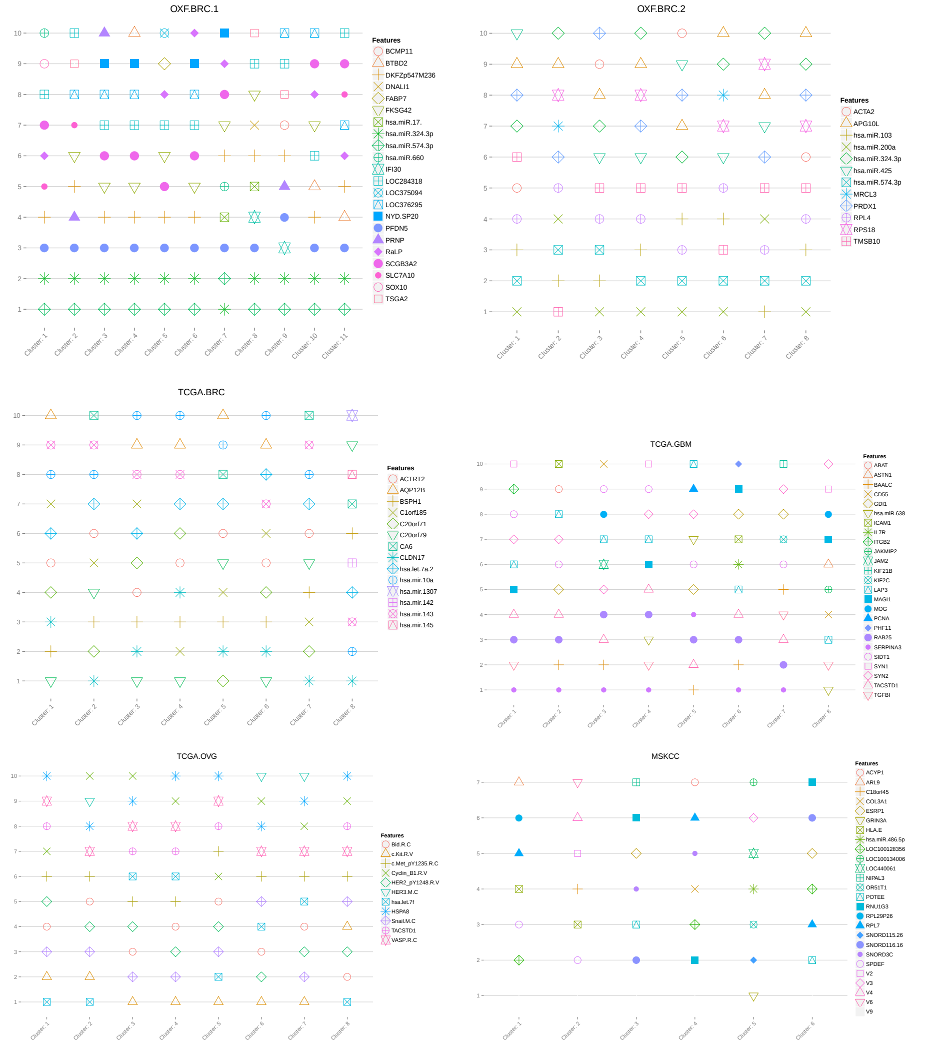

Figure 9 Box-plots of the TCGA.OVG: The box-plots of TCGA.OVG dataset were calculated on the multi-view clustering results obtained with the matrix factorization approach in semi-supervised mode. For space and clarity reasons, the box-plots of patients were drawn only on the features with the highest variance between the centroids of different clusters.

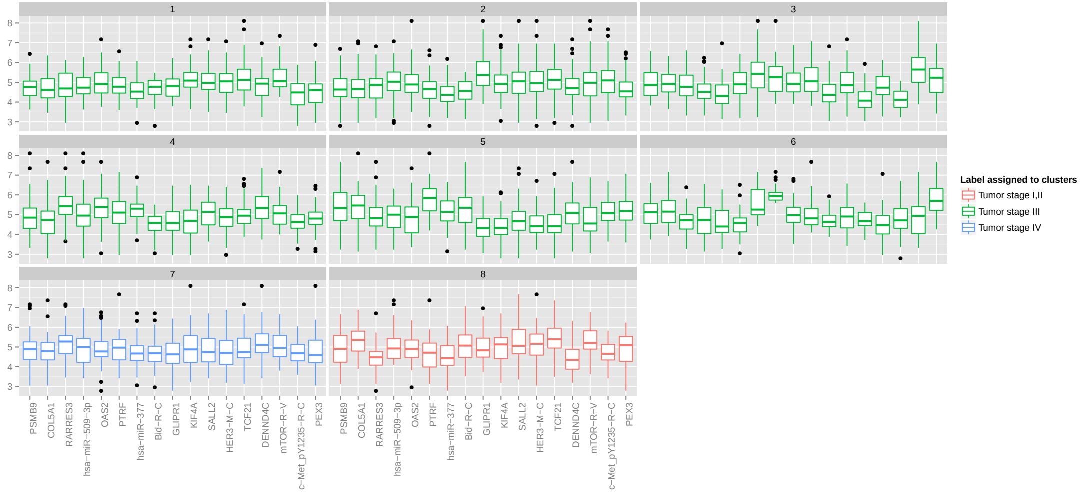

Figure 10 Box-plots of the MSKCC.PRCA: The box-plots of MSKCC.PRCA dataset were calculated on the multi-view clustering results obtained with the matrix factorization approach in semi-supervised mode. For space and clarity reasons, the box-plots of patients were drawn only on the features with the highest variance between the centroids of different clusters.

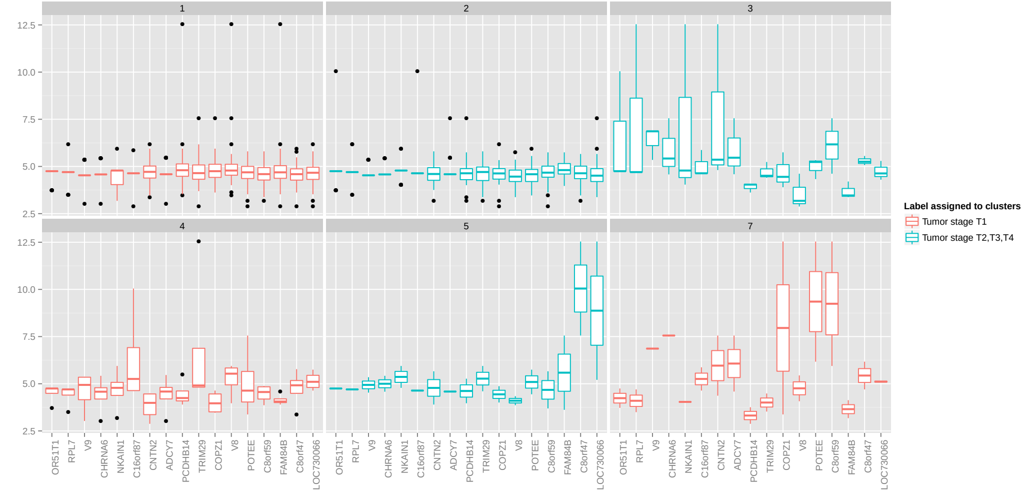

Figure 11 Box-plots of the OXF.BRCA.1 and OXF.BRCA.2: The box-plots of OXF.BRCA.1 and OXF.BRCA.2 datasets were calculated on the multi-view clustering results obtained with the matrix factorization approach in semi-supervised mode. For space and clarity reasons, the box-plots of patients were drawn only on the features with the highest variance between the centroids of different clusters.

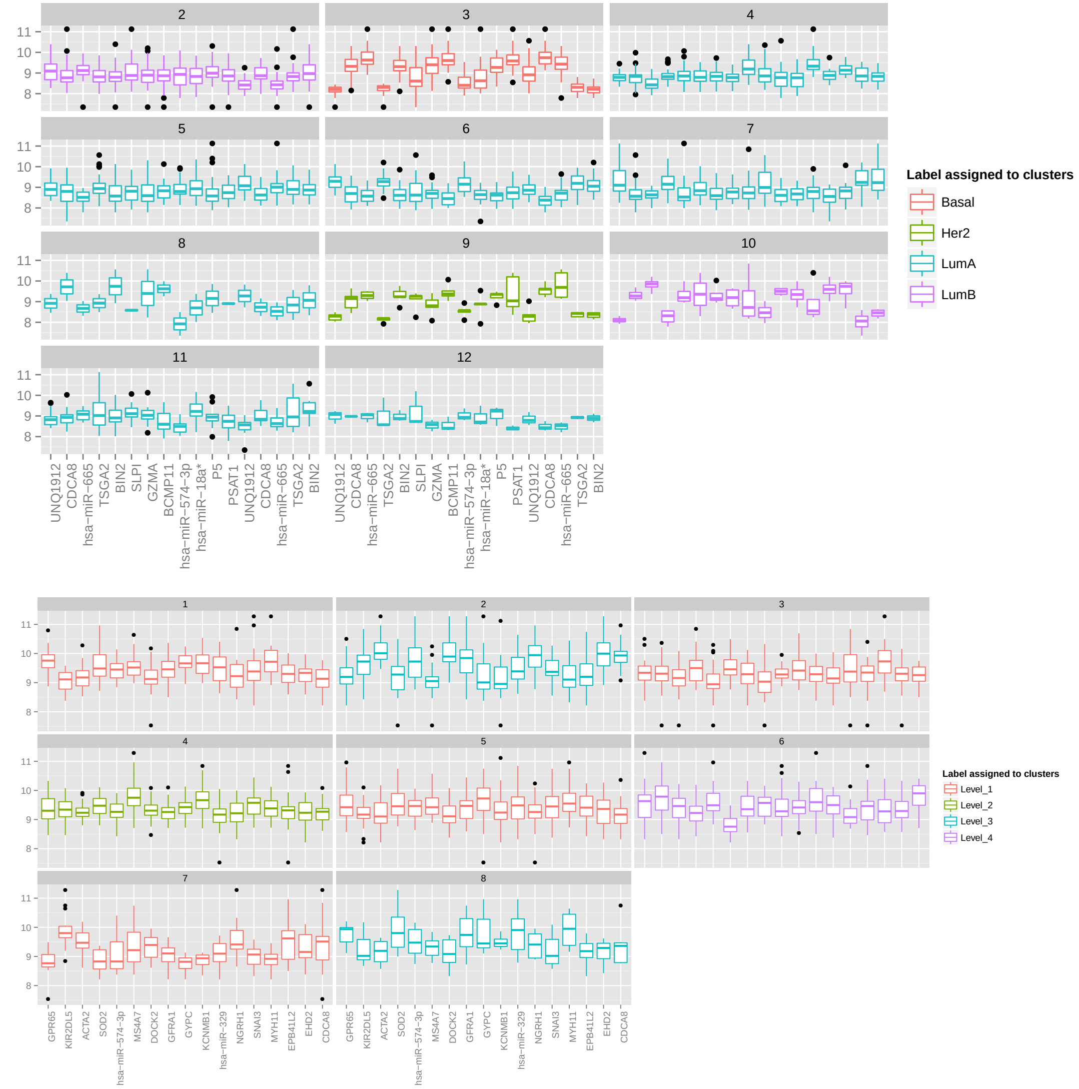

Figure 12 Box-plots of the TCGA.BRCA and TCGA.GBM: The box-plots of TCGA.BRCA and TCGA.GMB datasets were calculated on the multi-view clustering results obtained with the matrix factorization approach in semi-supervised mode. For space and clarity reasons, the box-plots of patients were drawn only on the features with the highest variance between the centroids of different clusters.

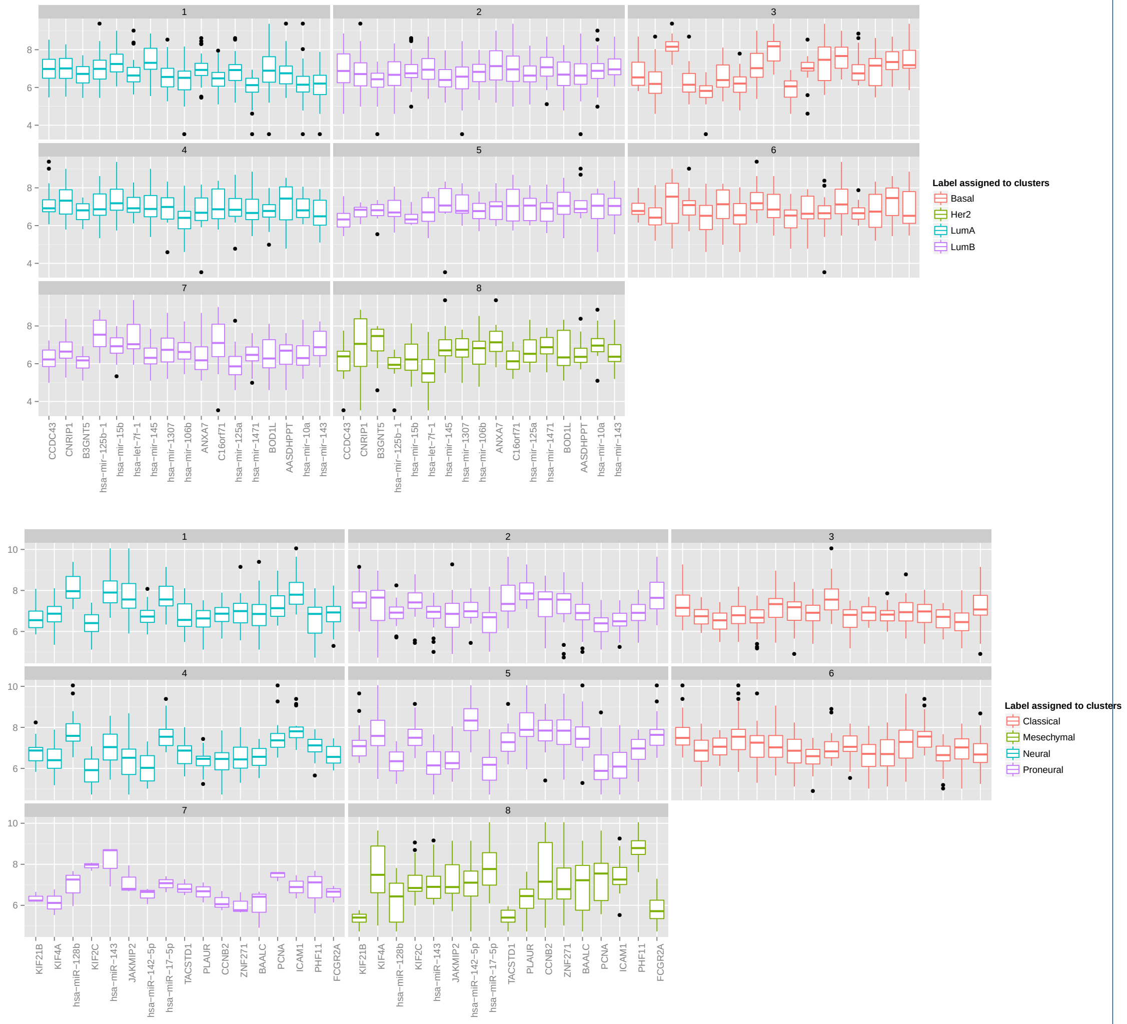

Validation Results

The method as been compared with classical single view clustering algorithms, early and intermediate integration approach.

We calculated classification error and normalized mutual information (NMI) for each method, between each clustering results and real patient classification.

Given two clustering solutions Cl1 and Cl2 NMI compute the mutual information between the two clustering normalized by the cluster entropies.

Because we know how patients are categorized we compute NMI between clustering results and real patient classifications.

	TCGA.BRCA	Algorithm	Error	NMI
Single View	All Feature	Ward	26,49%	41%
		Kmeans	29,14%	40%
		Pamk	23,18%	43%
	Selected Prototype	Ward	30,49%	41%
		Kmeans	31,79%	34%
		Pamk	23,18%	43%
Multi-View	Early Integration	Tw-kmeans	44,37%	43%
	Our method (unsupervised)	MF	*26,64%*	*37%*
	Our method (semi-supervised)	MF	*5,27%*	*70%*
	Intermediate Integration (all feat)	SNF	26,00%	38%
	Intermediate Integration (our feat)	SNF	32,00%	27%

	TCGA.OV	Algorithm	Error	NMI
Single View	All Feature	Ward	23,51%	3%
		Kmeans	22,04%	3%
		Pamk	20,89%	4%
	Selected Prototype	Ward	22,80%	3%
		Kmeans	25,76%	3%
		Pamk	21,02%	4%
Multi-View	Early Integration	Tw-kmeans	18,84%	4%
	Our method (unsupervised)	MF	*20,00%*	*8%*
	Our method (semi-supervised)	MF	*1,50%*	*44%*
	Intermediate Integration (all feat)	SNF	20,00%	4%
	Intermediate Integration (our feat)	SNF	20,50%	3%

	MSKCC.PRCA	Algorithm	Error	NMI
Single View	All Feature	Ward	37,90%	3%
		Kmeans	38,64%	2%
		Pamk	37,50%	3%
	Selected Prototype	Ward	37,50%	3%
		Kmeans	35,23%	4%
		Pamk	37,50%	3%
Multi-View	Early Integration	Tw-kmeans	27,27%	3%
	Our method (unsupervised)	GLI	*33,20%*	*10%*
	Our method (semi-supervised)	MF	*1,00%*	*72%*
	Intermediate Integration (all feat)	SNF	40,00%	0%
	Intermediate Integration (our feat)	SNF	36,98%	2%

	TCGA.GBM	Algorithm	Error	NMI
Single View	All Feature	Ward	17,96%	58%
		Kmeans	22,16%	56%
		Pamk	29,34%	46%
	Selected Prototype	Ward	18,96%	58%
		Kmeans	17,77%	57%
		Pamk	29,34%	46%
Multi-View	Early Integration	Tw-kmeans	57,49%	46%
	Our method (unsupervised)	MF	*26,00%*	*41%*
	Our method (semi-supervised)	MF	*7,78%*	*70%*
	Intermediate Integration (all feat)	SNF	24,00%	45%
	Intermediate Integration (our feat)	SNF	21,01%	43%

	OXF.BRCA.1	Algorithm	Error	NMI
Single View	All Feature	Ward	24,83%	32%
		Kmeans	22,39%	31%
		Pamk	22,86%	32%
	Selected Prototype	Ward	23,38%	32%
		Kmeans	20,87%	34%
		Pamk	22,89%	32%
Multi-View	Early Integration	Tw-kmeans	25,87%	32%
	Our method (unsupervised)	MF	*26,00%*	*41%*
	Our method (semi-supervised)	GLI	*7,00%*	*62%*
	Intermediate Integration (all feat)	SNF	*24,00%*	29%
	Intermediate Integration (our feat)	SNF	*28,00%*	23%

	OXF.BRCA.2	Algorithm	Error	NMI
Single View	All Feature	Ward	49,76%	16%
		Kmeans	51,24%	16%
		Pamk	50,75%	16%
	Selected Prototype	Ward	48,76%	16%
		Kmeans	50,75%	17%
		Pamk	50,75%	16%
Multi-View	Early Integration	Tw-kmeans	48,76%	16%
	Our method (unsupervised)	MF	*33,00%*	*33%*
	Our method (semi-supervised)	MF	*15,23%*	*59%*
	Intermediate Integration (all feat)	SNF	51,00%	13%
	Intermediate Integration (our feat)	SNF	49,34%	13%
