# Supplementary material for: MVDA: a multi-view genomic data integration methodology
Source: BMC Bioinformatics. 2015 Aug 19;16:261. doi: 10.1186/s12859-015-0680-3 (PMC4539887; doi:10.1186/s12859-015-0680-3)
Supplement: Additional file 3 — It contains the gene symbols and description for all shared genes between the tree breast cancer datasets highlighted by the analysis. (DOCX 14 kb) [file 12859_2015_680_MOESM3_ESM.docx]

List of genes shared between the three breast cancer datasets.

| **Gene Symbol** | **Description** |
| --- | --- |
| CCNE2 | cyclin E2 |
| CDC42EP1 | CDC42 effector protein (Rho GTPase binding) 1 |
| BLM | Bloom syndrome, RecQ helicase-like |
| CHST2 | carbohydrate (N-acetylglucosamine-6-O) sulfotransferase 2 |
| ALDH1A2 | aldehyde dehydrogenase 1 family, member A2 |
| ADH1B | alcohol dehydrogenase 1B (class I), beta polypeptide |
| CDC7 | cell division cycle 7 |
| ABCC12 | ATP-binding cassette, sub-family C (CFTR/MRP), member 12 |
| TYMS | thymidylate synthetase |
| ANG | angiogenin, ribonuclease, RNase A family, 5 |
| CKS1B | CDC28 protein kinase regulatory subunit 1B |
| COL9A3 | collagen, type IX, alpha 3 |
| BAI2 | [-](http://biodbnet.abcc.ncifcrf.gov/dbInfo/faq.php#data5) |
| C6orf150 | Mab-21 domain containing 1 |
| CDCA8 | cell division cycle associated 8 |
| ANLN | anillin, actin binding protein |
| CBR3 | carbonyl reductase 3 |
| ABCG1 | ATP-binding cassette, sub-family G (WHITE), member 1 |
| AZU1 | azurocidin 1 |
| CDKN1B | cyclin-dependent kinase inhibitor 1B (p27, Kip1) |
| CEACAM1 | carcinoembryonic antigen-related cell adhesion molecule 1 (biliary glycoprotein)] [Gene Type: protein-coding] |
| ANXA8 | annexin A8 |
| KIF2C | kinesin family member 2C |
| CHI3L2 | chitinase 3-like 2 |
| APOBEC3B | apolipoprotein B mRNA editing enzyme, catalytic polypeptide-like 3B |
| ALCAM | activated leukocyte cell adhesion molecule |
| CMA1 | chymase 1, mast cell |
| COL22A1 | collagen, type XXII, alpha 1 |
| C6orf15 | chromosome 6 open reading frame 15 |
| C1orf21 | chromosome 1 open reading frame 21 |
| CECR5 | cat eye syndrome chromosome region, candidate 5 |
| ADRA2B | adrenoceptor alpha 2B |
| ACRV1 | acrosomal vesicle protein 1 |
| ACO2 | aconitase 2, mitochondrial |
| CHL1 | cell adhesion molecule L1-like |
| CACNG4 | calcium channel, voltage-dependent, gamma subunit 4 |
| CA1 | carbonic anhydrase I |
| C17orf28 | HID1 domain containing |
| AMBP | alpha-1-microglobulin/bikunin precursor |
| ALDH3B2 | aldehyde dehydrogenase 3 family, member B2 |
| CHD1L | chromodomain helicase DNA binding protein 1-like |
| ANPEP | alanyl (membrane) aminopeptidase |
| ALDOC | aldolase C, fructose-bisphosphate |
| BMP5 | bone morphogenetic protein 5 |
| ADCY5 | adenylate cyclase 5 |
